# Normalization of Fetal Cerebral and Hepatic Iron by Parental Iron Therapy to Pregnant Rats with Systemic Iron Deficiency without Anemia

**DOI:** 10.3390/nu16193264

**Published:** 2024-09-27

**Authors:** Annette Burkhart, Kasper Bendix Johnsen, Tina Skjørringe, Asbjørn Haaning Nielsen, Lisa Juul Routhe, Sandra Hertz, Lisbeth Birk Møller, Lars Lykke Thomsen, Torben Moos

**Affiliations:** 1 Neurobiology Research and Drug Delivery (NRD), Department of Health Science and Technology, Aalborg University, 9260 Gistrup, Denmark; abl@hst.aau.dk (A.B.); tinaskj@gmail.com (T.S.); ljrouthe@gmail.com (L.J.R.);; 2 Section of Biotherapeutic Engineering and Drug Targeting, Department of Health Technology, Technical University of Denmark, 2800 Lyngby, Denmark; kasperbendixjohnsen@gmail.com; 3 Division of Water and Soil, Department of the Built Environment, Aalborg University, 9220 Aalborg, Denmark; ahn@build.aau.dk; 4 Center for Applied Human Genetics, Kennedy Center, Copenhagen University Hospital, 2600 Glostrup, Denmark; lisbeth.birk.moeller@regionh.dk; 5 Pharmacosmos A/S, 4300 Holbæk, Denmark; llt@pharmacosmos.com

**Keywords:** copper, ferroportin, hepcidin, iron, iron deficiency, zinc

## Abstract

Background/Objectives: Iron (Fe) is a co-factor for enzymes of the developing brain necessitating sufficient supply. We investigated the effects of administering ferric derisomaltose/Fe isomaltoside (FDI) subcutaneously to Fe-deficient (ID) pregnant rats on cerebral and hepatic concentrations of essential metals and the expression of iron-relevant genes. Methods: Pregnant rats subjected to ID were injected with FDI on the day of mating (E0), 14 days into pregnancy (E14), or the day of birth (postnatal (P0)). The efficacy was evaluated by determination of cerebral and hepatic Fe, copper (Cu), and zinc (Zn) and gene expression of ferroportin, hepcidin, and ferritin H + L in pups on P0 and as adults on P70. Results: Females fed an ID diet (5.2 mg/kg Fe) had offspring with significantly lower cerebral and hepatic Fe compared to female controls fed a standard diet (158 mg/kg Fe). Cerebral Cu increased irrespective of supplying a standard diet or administering FDI combined with the standard diet. Hepatic hepcidin mRNA was significantly lower following ID. Cerebral hepcidin mRNA was hardly detectable irrespective of iron status. Conclusions: In conclusion, administering FDI subcutaneously to ID pregnant rats on E0 normalizes fetal cerebral and hepatic Fe. When applied at later gestational ages, supplementation with additional Fe to the offspring is needed to normalize cerebral and hepatic Fe.

## 1. Introduction

According to WHO, iron (Fe) deficiency (ID) affects approximately 20% of the population worldwide, which is equal to 1.4–2.0 billion people. ID is especially common in females during the reproductive age and in young children [1]. In developing countries, it is estimated that 52% of pregnant women have anemia mainly caused by ID [1]. Although ID mostly occurs in developing countries, industrialized countries certainly also face the challenges of ID. The prevalence of ID in female teenagers in the UK is approximately 21% [2]. During pregnancy, the demand for Fe in both mother and fetus increases, and ID occurring with anemia is particularly common during late pregnancy. In a study from the US, the frequency of ID with anemia in the first, second, and third trimesters was 2%, 8%, and 27%, respectively [3].

Due to the serious consequences of fetal ID, health authorities recommend supplemental Fe during pregnancy. A series of products for oral supplementation are available, but orally administered Fe has low intestinal uptake. A standard oral treatment includes solid or liquid iron supplement preparations consisting of ferrous salts such as ferrous sulfate, ferrous fumarate, or ferrous gluconate plus adjoining liquid supplements. Studies in intestinal cells in vitro reveal that as little as 30–40% of seven Fe substitution products based on the ferrous salts mentioned above undergo transcellular transport [4]. Additionally, the uptake and transport of Fe through the intestinal wall are influenced by the composition of the diet. Meat and ascorbic acid ease the Fe uptake and transport, whereas polyphenols, phytates, and calcium lead to a reduction [4]. Only 48% of pregnant women with ID and anemia supply their diet with oral Fe. The poor compliance in combination with poor Fe intestinal absorption can nonetheless still lead to sustained ID [5]. Therefore, parenteral Fe may be more efficient, as compliance is increased and diminished Fe uptake in the gut is avoided.

In the central nervous system (CNS), Fe is a co-factor for a variety of enzymes needed for the formation of proteins and lipids vital for normal cellular function. Fe is essential for cell division [6], including neuronal precursors of the developing brain, hence making gestational ID a serious challenge. Supporting this notion, severe prenatal ID in humans increases the risks of developing impaired motor performance, poor cognitive performance, and problems with social and attentional behavior [7]. 

Sufficient Fe supply to the developing brain is ensured by the substantial expression of transferrin receptors on neuronal precursor cells and brain capillary endothelial cells that form the blood–brain barrier [8,9]. The handling of Fe by the cells of the CNS is further safeguarded by the expression of ferritin that prevents excess free Fe from exerting deleterious effects [10]. The Fe levels may also be regulated via efflux across the cellular membrane via the expression of the ferrous Fe exporter ferroportin [11,12]. However, the plasticity of this protein in the brain in response to changes in the Fe levels of the CNS has been examined only in a few studies (e.g., [13]). The expression level of ferroportin protein is post-translationally regulated by hepcidin, a hepatic hormone whose circulatory level proportionally increases in response to increasing circulatory Fe and the reverse in ID [14]. Studies almost failed to detect the expression of hepcidin in the normal CNS using mRNA analyses, and the hepcidin peptide concentration in the normal CNS is extremely low [15,16]. 

ID also affects the metabolism of other metals, mainly copper (Cu) and zinc (Zn) [17,18,19]. Cu is a co-factor for many proteins, among others, dopamine beta monooxygenase and superoxide dismutase, which are essential for the normal function of the CNS. Zn functions as a co-factor for an estimated 3000 human proteins and dysfunctional Zn-signaling is associated with serious conditions in the CNS like Alzheimer’s disease, cardiovascular disease, diabetes, and cancer [20]. Hence, any adverse effect on the metabolism of these metals is serious. 

Ferric derisomaltose/iron isomaltoside (FDI) (Pharmacosmos, Holbæk) is a Fe–carbohydrate matrix formulation that has been marketed since 2010. It consists of Fe and a carbohydrate moiety with a low immunological activity where Fe is tightly bound in a matrix structure, which allows for fast administration of high Fe doses even by a single injection [21,22,23,24]. Here, we studied the hypothesis that FDI is efficacious with respect to reversing Fe depletion in the developing brain. For this purpose, we established a rat model of gestation, where FDI was administered to pregnant dams on three distinct time points (E0, E14, or P0). The efficacy was evaluated by determination of the brain and liver Fe concentration of the pups on P0 and P70 as well as the concentration of Cu and Zn. We also studied the ferroportin, hepcidin, and ferritin H + L genes, and the impact of FDI treatment on their expression.

## 2. Materials and Methods

### 2.1. Gestational Fe Deficiency

Wistar rats were housed in cages at the Animal Department of Aalborg University Hospital under constant temperature and humidity conditions and a 12 h light/dark cycle with free access to food and water. The Danish Experimental Animal Inspectorate under the Ministry of Food and Agriculture approved all handling of the rats in this study (permission no. 2013-15-2934-00776). Seventy-six female rats were fed a normal diet (C1000, Altromin, Lage, Germany) until postnatal (P) day 42 (P42). Sixty-two of the rats were then switched to an Fe-deficient diet with Fe content of 5.2 mg/kg (C1038, Altromin, DE, USA) for six weeks until P84, whereas the remaining fourteen14 rats were kept on a normal diet. To analyze the Fe status of the female rats at the time of fertilization, rats fed the Fe-sufficient (n = 6) or Fe-deficient (n = 6) diet were euthanized equal to the time of conception (P84), and their hemoglobin and liver content of Fe, Cu, and Zn measured. The presence of ID was identified as a significant lowering of the hepatic Fe concentration, and additional ID with anemia was verified by measuring the hemoglobin concentration.

**Figure 1:**
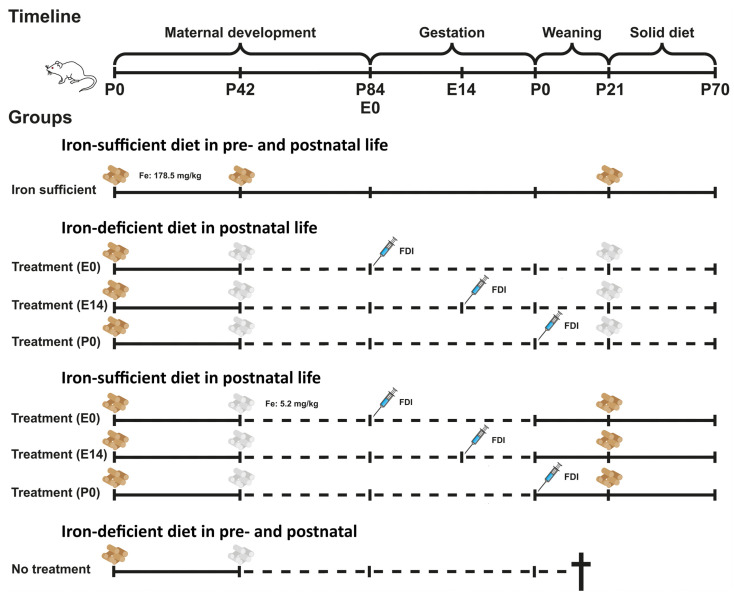
Schematic representation of the gestational ID model. The offspring was examined on days P0 and P70. Initially, the pregnant rats were divided into four different groups; Fe-sufficient diet, treatment on E0, treatment on E14, or treatment on P0. Twelve pups of either sex were euthanized on day P0 and collected from the four groups. From P0, the mother rats were either maintained on the ID diet with an Fe content of 5.2 mg/kg (dashed lines) or changed to a diet replete in Fe with content of 178.5 mg/kg (solid lines). The pups were weaned from P21 and kept on the same diet as their respective mothers until euthanasia on P70 (n = 12 per group). †, the passing of the neonatal pubs when not receiving iron. E, Embryonic; FDI, Ferric derisomaltoside; Fe, Iron; P, Postnatal.

The non-euthanized aged P84 female rats were fertilized by male rats fed a normal diet. For Fe supplementation, the mothers fed the diet with a low Fe content were injected subcutaneously with ferric derisomaltose (FDI; formerly iron isomaltoside 1000) (Pharmacosmos, Holbæk, Danmark) at a dose of 80 mg/kg on day E0 (the day of conception) (n = 16), day E14 (E14) (n = 16), or the day of giving birth, day P0 (P0) (n = 16). From P0, the mother rats were either maintained on the ID diet with an Fe content of 5.2 mg/kg (Figure 1, dashed lines) or changed to a diet replete in Fe with a content of 178.5 mg/kg (Figure 1, solid lines). We also defined a separate group (no treatment) consisting of pregnant rats maintained on the ID diet without Fe supplementation (n = 8). However, the offspring was not vital and would typically die within two to three weeks after birth for reasons justifiable for euthanasia.

The offspring were examined on days P0 and P70 (Figure 1). Twelve pups of either sex were euthanized on day P0. From P0 onwards, the mother rats were either maintained on the ID diet with an Fe content of 5.2 mg/kg (Figure 1, dashed lines) or changed to a diet replete in Fe with a content of 178.5 mg/kg (Figure 1, solid lines). Male pups were weaned from P21 and kept on the same diet as their respective mothers until euthanasia on P70 (n = 12 per group) (Figure 1). The pups weaned from P21 were all males to prevent breeding.

The experiments were run in duplicate, and the data presented are based on collected blood and tissue from both experiments. For tissue collection from the P0 and P70 rats, they were deeply anesthetized with a subcutaneous injection of 0.5 mL/10 g body weight of Hypnorm/Dormicum (fentanyl/fluanisone mixed with midazolam). Subsequently, the chest was opened, and a blood sample was collected from the P0 rats for measurement of the hemoglobin concentration (HemoControl, HaemoMedtec, Ikast, Danmark). Five ml blood of the P70 rats was then collected in a heparinized syringe followed by transcardial perfusion of the rats with heparinized saline to remove blood from the vasculature that could otherwise interfere with the measurements of metal content of the brain and liver. The heparinized blood samples were analyzed for various hematological parameters at the Department of Clinical Biochemistry, Aalborg University Hospital, Denmark using a standard procedure for measures of human patients’ blood with a main emphasis on the erythron.

### 2.2. Metal Analyses

The brain stem and liver of P0 and P70 rats were dissected and used for the detection of metals, together with heparinized blood taken from the pregnant mothers. The Fe content of the brain stem is representative of the concentration of Fe in the rat CNS [25]. The concentrations of Fe, Cu, and Zn were measured by inductively coupled plasma-optical emission spectrometry (ICP-OES) [26]. The tissue samples were freeze-dried, homogenized, and transferred to fluoropolymer-lined ceramic digestion vessels. The samples were then digested using microwave-assisted acid digestion with 8 mL concentrated nitric acid for 10 min at 1200 W. The microwave reaction system was an Anton Paar Multiwave 3000 equipped with 16 digestion vessels of 100 mL each (Anton Paar GmbH, Graz, Austria). Cooled digestates were diluted to 50 mL with type 1 ultrapure water (PURELAB Ultra, Elga LabWater (Kruger Aquacare), Glostrup, Denmark), transferred to plastic flasks, and allowed to settle before being analyzed for metals. Metal concentrations were measured axially using a Thermo iCap 6300 DuoView ICP-OES instrument (Thermo Scientific, Cambridge, UK). The plasma and auxiliary gas flows were set at 12 L/min and 1.0 L/min, respectively. The forward power of the ICP-OES was 1.15 kW.

The samples were introduced into the plasma via a cyclonic spray chamber equipped with a concentric glass nebulizer. The nebulization gas pressure was 0.2 MPa, and the sample uptake rate was 2 mL/min. Matrix-matched multi-element external standards were used for calibration. The standards were prepared by the National Institute of Standards and Technology (NIST) traceable single-element standards of 1000 µg/mL (PlasmaCAL, SCP Science, Quebec, QC, Canada). Two standards and one blank per element were used for the calibration (i.e., 0.0, 20, 200 µg/L). Each element was quantified using two or three emission lines—each measured in triplicate (Fe: 238.204 nm, 239.562 nm, 259.940 nm; Cu: 324.754 nm, 327.396 nm; Zn: 206.200 nm, 213.856 nm). The integration time was set at 5 s for emission lines above 243 nm and 15 s for the remaining emission lines. Yttrium was supplemented to all standards and samples and used as an internal standard (measured at 371.030 nm) to compensate for matrix effects.

### 2.3. Gene Expression Analyses

Brain stems and livers were examined for expression of ferroportin and hepcidin using the following primers: Ferroportin (reference sequence: NM_133315): forward primer CCCTGCTCTGGCTGTAAAAG, reverse primer GCACAGGTGGGTTCTTGTTC); hepcidin (reference sequence: NM_053469): forward primer GGCAACAGACGAGACAGACT, reverse primer AACAGAGACCACAGGAGGAA; ferritin H (reference sequence: NM_031329.2). Forward primer, CTGACTATGCGGAAAGAGTCGACAG. Reverse primer, AGAGGAATCTCCTGGGCTACTTCAG); and ferritin L (reference sequence: NM_031701). Forward primer, CTACAGGCTCTTGTGAGGACTTGAC. Reverse primer, AGTAGGAACTGTTAGCGGCAGTTTG). The RNA was extracted using the GeneJET RNA Purification Kit and treated with DNase I enzyme according to the manufacturer’s protocol. One hundred nanograms of each DNA-free RNA sample served as a template for RT-qPCR. cDNA synthesis was performed using Revert Aid Premium First Strand cDNA Synthesis Kit, and 2.5 ng cDNA and 10 pmol of each primer used as listed for each qPCR reaction together with the Maxima™ SYBR Green qPCR Master Mix. We performed the PCRs according to the MIQE guidelines [27] and analyzed each sample in triplicates. Non-reverse transcribed RNA and water served as negative controls. Beta-actin was used as a housekeeping gene for normalization purposes, and Stratagene Mx 3000P^TM^ QPCR System (Agilent Technologies, Hørsholm, Denmark) was used for RT-qPCR analysis. The PCR conditions were 95 °C for 10 min, followed by 40 cycles of 95 °C for 30 s, 60 °C for 30 s, and 72 °C for 30 s. The relative quantities of DNA were calculated in the analyzed samples by the Pfaffl method [28] using the Fe-sufficient group as the calibrator. We found no evidence of iron status affecting the expression of the housekeeping gene b-actin in the analyzed tissues.

### 2.4. Statistics

All data are presented as mean ± standard deviation (SD). n values refer to the number of animals included in the analyses. All datasets were initially analyzed for potential outliers using a Grubbs test and subsequently tested for Gaussian distribution and equal variances using the Brown–Forsythe test to evaluate if the data were normally distributed. When normally distributed, the data were analyzed using a one-way ANOVA with Dunnett’s multiple comparisons test to determine statistical significance in the Fe-sufficient group versus the different treatment groups. When normality could not be assumed, we employed the non-parametric Kruskal–Wallis test with Dunn’s multiple comparison post hoc test instead. All calculations were performed in GraphPad Prism version 8-9 software and *p* values < 0.05 were considered statistically significant.

## 3. Results

We established a model of gestational ID to determine the impact on the offspring after treatment with FDI. The pregnant dams fed an ID diet presented with a significant decrease in the hepatic Fe stores, whereas other parameters like total body weight, plasma Fe, hemoglobin, and liver concentration of Cu and Zn were comparable to those of the controls (Table 1). 

**Figure 2:**
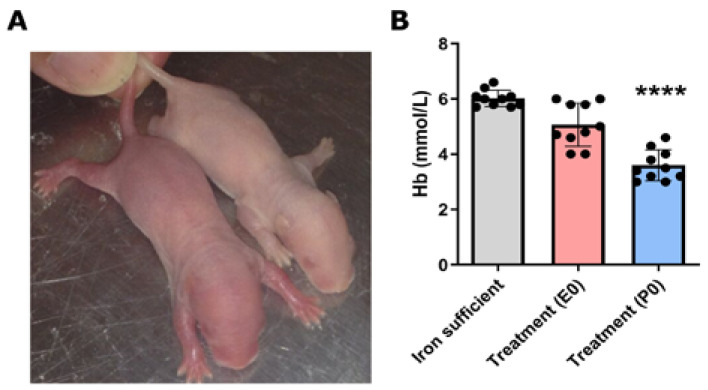
Characteristics of newborn offspring after maternal ID. (**A**) Pups of mothers fed a Fe-sufficient diet presented a healthy skin color (**left**), whereas those exposed to ID had a phenotype characterized by pale skin. (**right**) (**B**) Hemoglobin levels measured in the blood of newborn pups being exposed either to an Fe-sufficient or ID diet and treated with ferric derisomaltoside (FDI) either on gestational day E0 or P0. Data are presented as mean ± SD with each sample marked as individual black dots (n = 7). The ordinary one-way ANOVA and Dunnett’s multiple comparison post hoc test were used to test the statistical significance of individual differences with the Fe-sufficient control group as a reference point. **** *p* < 0.0001.

A sampling of the offspring immediately after birth (P0) revealed a clear change in phenotype with pups of ID mothers being characterized by a much paler skin tone compared to pups of ID-sufficient mothers (Figure 2A). Hemoglobin levels were reduced in the offspring of maternal treatment groups, with the pups of untreated mothers during gestation (P0) presenting the biggest decrease (Figure 2B).

**Figure 3:**
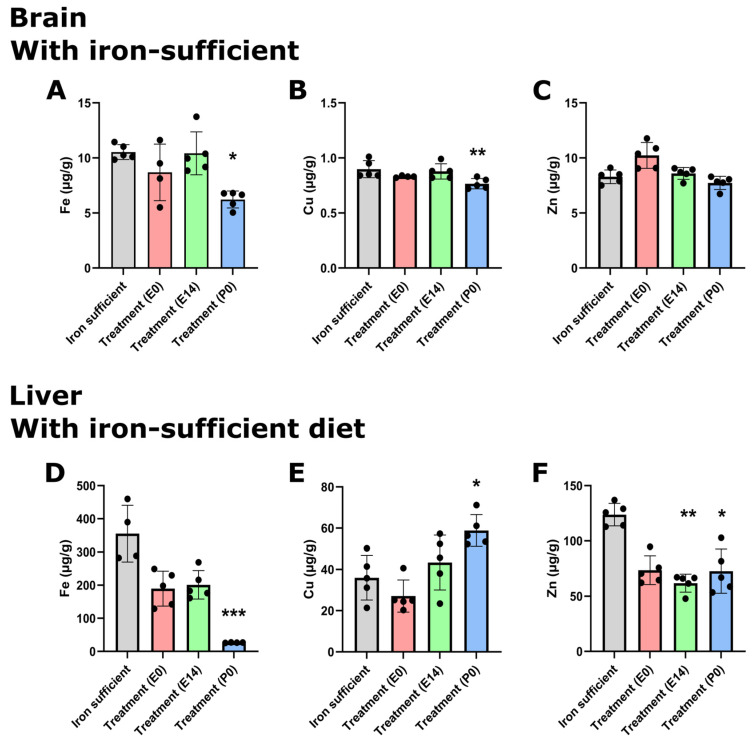
Metal composition of newborn brain and liver on P0 after ID and FDI treatment. (**A**) Cerebral Fe concentration of the pups after maternal feeding Fe-sufficient diet or ID diet added with the treatment of a single intramuscular injection of FDI on E0 or E14. The P0 treatment group represents untreated animals at this time point, as these pups were taken from mothers fed an ID diet without injection of FDI. (**B**) Cu and (**C**) Zn concentrations in the brain. Liver concentrations of Fe (**D**), Cu (**E**), and (**F**) Zn. As expected, the concentration of Fe decreases with reduced dietary access to Fe. This is particularly evident in the liver where a tendency is seen towards a reverse increase in Cu with increasing time-period deprived in Fe, and with a tendency to lower Zn with extended ID. Data are presented as mean ± SD with each sample marked as individual black dots (n = 4–8). The non-parametric Kruskal–Wallis test and Dunn’s multiple comparison post hoc test were used to test the statistical significance of individual differences with the Fe-sufficient control group as a reference point. * *p* < 0.05, ** *p* < 0.01, *** *p* < 0.001.

The offspring were further analyzed for the concentration of three important transition metals, i.e., Fe, Cu, and Zn, on P0. The cerebral Fe concentration was normalized after treatment with FDI (treatment groups E0 and E14). The treatment group (P0) had a statistically significant decrease (Figure 3A). Cu and Zn concentrations were not changed to any significant degree except for a small but statistically significant reduction in Cu concentration observed in the P0 group (Figure 3B,C). The liver Fe concentration was reduced in all treatment groups (Figure 3D). The Fe concentrations measured in the offspring of the E0 and E14 treatment groups were higher than those of the pups whose mothers were otherwise supposed to receive treatment at birth (Treatment (P0)) (Figure 3D). In the livers of the P0 group, the depletion of Fe was adjoined with an increase in Cu (Figure 3E) and a decrease in Zn (Figure 3F).

We then evaluated the offspring at P70 to investigate how maternal ID and subsequent treatment with FDI affected the metal composition of the brain and liver in early adulthood. Half of the animals were kept on an ID diet in their postnatal life, while the other half received a Fe-sufficient diet from P21 to decipher the chronic impact that was imposed directly by treatment with FDI versus that imposed by merely changing the diet to be Fe-sufficient. The animals fed the Fe-sufficient diet after birth presented with growth and hematological parameters that largely mirrored that of Fe-sufficient controls (Table 2). However, the animals that were kept on an ID diet in postnatal life showed several distinct changes. Of note, these animals were generally characterized by a significantly lower total body weight, hemoglobin (HGB) concentration, mean corpuscular volume (MCV), and mean cell hemoglobin concentration (MCHC). Furthermore, large increases in reticulocyte and thrombocyte count were also observed. Together these physiological parameters were suggestive of persistent ID that was not corrected chronically by the single dose of FDI administered to the respective mothers during gestation (Table 2).

**Figure 4:**
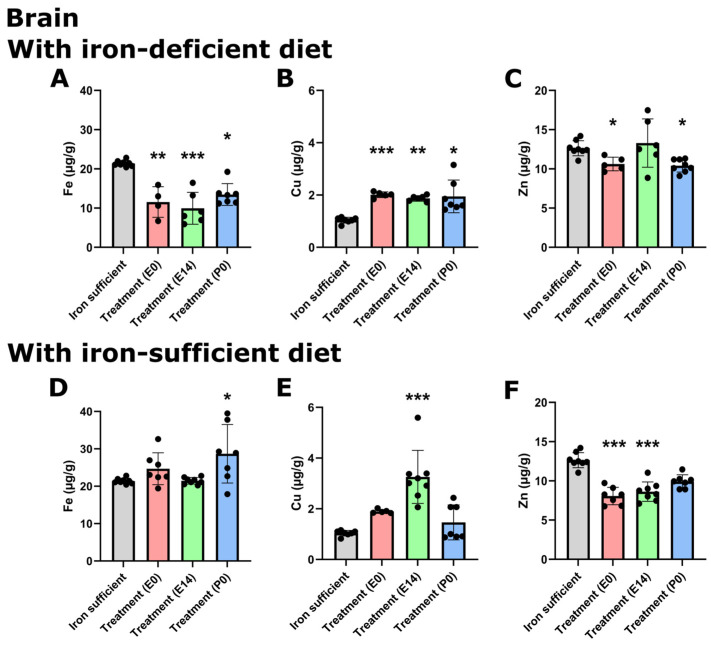
Cerebral metal composition on P70 after maternal feeding an Fe-sufficient diet or an ID diet added with treatment with a single injection of FDI on E0, E14, or P0. The data are presented with the Fe- sufficient control group (gray column) is used both in the top and bottom panels to improve the visual appearance and to enable pattern-based interpretations as a function of postnatal diet. In the brains of P70 rats, where mothers were fed the ID diet added with FDI on E0, E14, or P0, and the pups after weaning continued on the ID diet P21, Fe (**A**) is expectedly lower in the brain with Cu (**B**) rising to significantly higher levels. (**C**) Zn is slightly lower in two out of three groups receiving the ID diet. (**D**,**F**) Examining the brains of P70 fed a normal Fe-containing diet from P21 reveals increases in both Fe (**D**) and Cu (**E**) compared to those of rats only fed the control diet, and Zn slightly lower (**F**). * *p* < 0.05, ** *p* < 0.01, *** *p* < 0.001.

In animals fed an ID diet, the cerebral Fe concentration was significantly decreased in all treatment groups (Figure 4A), again pointing towards the fact that despite a single large Fe dose given during gestation, this cannot fulfill the cerebral Fe requirements chronically should ID persist. The lowering of the cerebral Fe concentration further coincided with a general increase in the brain Cu concentration (Figure 4B), while modest decreases in the Zn concentration were observed when mothers received FDI treatment on E0 and P0, the latter being the time point where mothers were otherwise supposed to receive treatment (Figure 4C). In the animals fed Fe-sufficient diets after weaning on P21, the brain Fe concentration was similar to that of the Fe-sufficient control, although with a modest increase observed in the animals with their mothers having received the FDI at P0 (Figure 4D). The Cu concentration was significantly increased in the E14 treatment group with the same tendency observed in the E0 treatment group (Figure 4E). In addition, the Zn concentration was generally lowered after FDI treatment, most prominently in the E0 and E14 treatment groups (Figure 4F).

**Figure 5:**
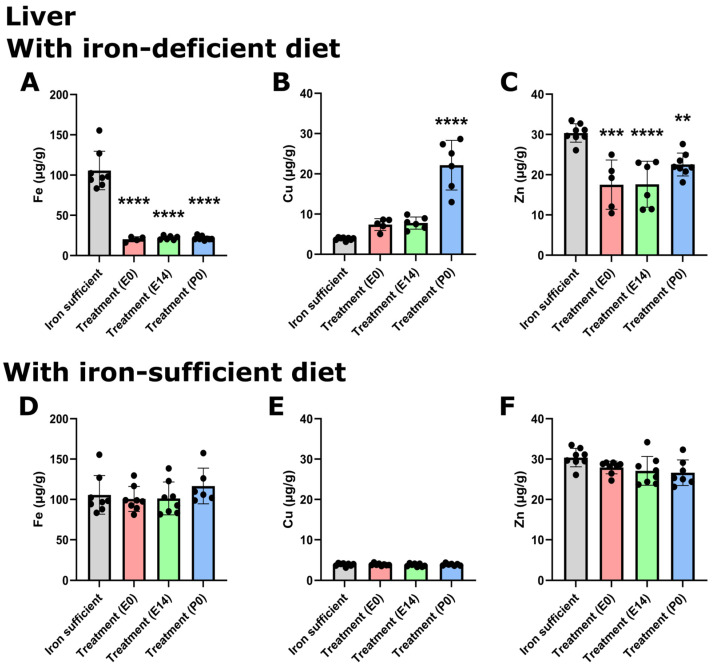
Hepatic metal composition on P70 after maternal feeding an Fe-sufficient diet or an ID diet added with treatment with a single injection of FDI on E0, E14, or P0. The data are presented with the Fe- sufficient control group (gray column) is used both in the top and bottom panels to improve the visual appearance and to enable pattern-based interpretations as a function of postnatal diet. Lower. In the livers of P70 rats, where mothers were fed the ID diet added with FDI on E0, E14, or P0, and the pups after weaning continued on the ID diet from P21, Fe (**A**) is significantly lower in the three groups compared to the liver of the rats of the Fe sufficiently fed control group (gray column). Cu (**B**) increases, and Zn (**C**) is lower. (**D**,**F**) Examining livers of P70 fed a normal Fe-containing diet from P21 reveals a slight increase in Fe (**D**), whereas Cu (**E**) is unchanged, and Zn (**F**) is slightly lower. Data are presented as mean ± SD with each sample marked as individual black dots (n = 4–8). The non-parametric Kruskal–Wallis test and Dunn’s multiple comparison post hoc test were used to test the statistical significance of individual differences with the Fe-sufficient control group as a reference point. ** *p* < 0.01, *** *p* < 0.001., **** *p* < 0.001.

When examining the livers of the same animals at P70, the liver Fe stores were severely depleted in rats fed the ID diet (Figure 5A), which correlated with an increase in Cu concentration (especially in the animals treated at P0) (Figure 5B), and a decrease in the liver Zn concentration (Figure 5C). As also observed in the brain, we found that the liver Fe concentration was restored when treated animals were fed the Fe-sufficient diet in their postnatal life from P21 (Figure 5D). The same was observed for the Cu concentration (Figure 5E), while small but statistically significant reductions in the Zn concentration were detected in animals of the E14 and P0 treatment groups (Figure 5F).

**Figure 6:**
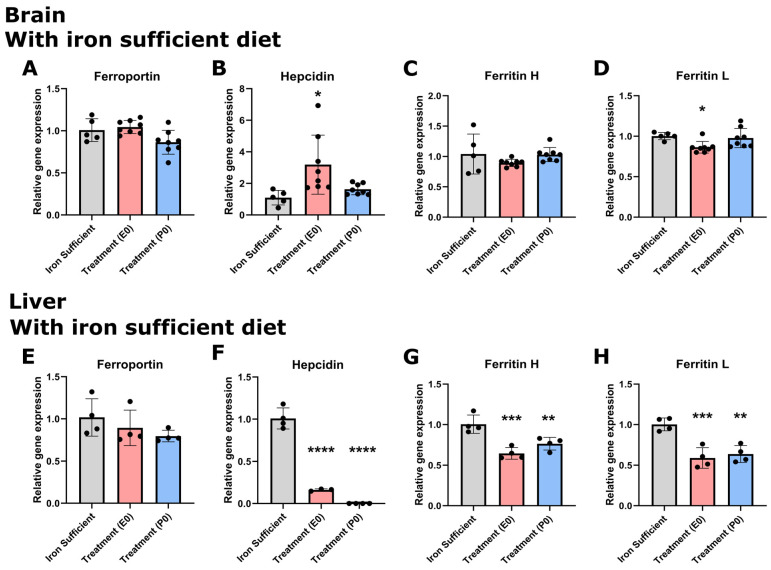
Ferroportin (**A**,**C**) and hepcidin (**B**,**D**) mRNA expression in CNS (**A**,**B**) and liver (**C**,**D**) of P0 rats. Ferroportin (**A**,**E**), hepcidin (**B**,**F**), and ferritin H (**C**,**G**) and L (**D**,**H**) mRNA expression in CNS and liver of P0 rats. A. Cerebral ferroportin is slightly lower in the P0 group, but unaltered in the liver (**E**). Hepcidin is generally slightly expressed in the CNS (**B**) compared to the liver (**F**) in normal-fed rats (see text). Following ID, the E0 group is significantly higher than both the control and the C group, but the general expression level is very low. In the liver, ID leads to significantly low expression of hepcidin in both E0 and P0 groups. (**C**,**D**,**G**,**H**), In the case of ferritin H and L, the expression in the CNS is slightly lower in the E0 group concerning ferritin L, and clearly lower in the livers of the ID groups. Data are presented as mean ± SEM (n = 5–8). * *p* < 0.05, ** *p* < 0.01, *** *p* < 0.001, **** *p* < 0.0001.

We next wanted to study the expression of ferroportin, hepcidin, and ferritin H + L in response to these dietary changes in cerebral and hepatic content. In P0 pups whose mothers were otherwise supposed to receive treatment at birth, a comparison between the different groups by P0 revealed that the cerebral expression of ferroportin was non-significant (Figure 6A). In the liver on P0, it was not possible to detect significant differences either among groups, but the tendency was clearly the gene expression of ferroportin being lower following ID (Figure 6E). The expression of ferroportin in the brain was overall approximately 150 times lower than that seen in the liver of respective experimental groups (typical CT values for ferroportin/ b-actin were determined as 23.7/21.4 in the liver and 18.0/28.4 in the brain).

The difference in expression level was more dramatic when comparing the expression of hepcidin in the brain versus the liver (typical CT values for hepcidin/ b-actin determined as 20.9/22.5 in the liver and 36.1/18.8 in the brain). Hence, hepcidin expression was extremely low in the brain of P0 pups, and in the normal liver approximately 8.5 million times higher compared to that of the normal brain. Considering the expression of hepcidin in the brain, the gene expression of hepcidin in the group receiving treatment on E0 proved significantly higher than both the control and the group with mother meant to have received treatment on P0, but it should also here be noted that the expression in the E0 was extremely low, not at least compared to the expression in the liver. In pups from both ID groups (E0, P0), the gene expression of liver hepcidin was significantly lower than in the controls (Figure 6F). The expression levels of the ferritin H and L subunits in the brain were much higher than those of ferroportin and hepcidin. The cerebral gene expression of the ferritin H and L subunits was almost unaltered following maternal ID, but the group E0 was slightly lower (Figure 6C,D). In the liver, ferritin H + L subunits were significantly lower in the pups from the ID groups (Figure 6G,H). Noteworthy, whereas the gene expression of ferritin H in the brain and liver was almost identical, the expression of ferritin L was approximately ten times higher in the liver. 

**Figure 7:**
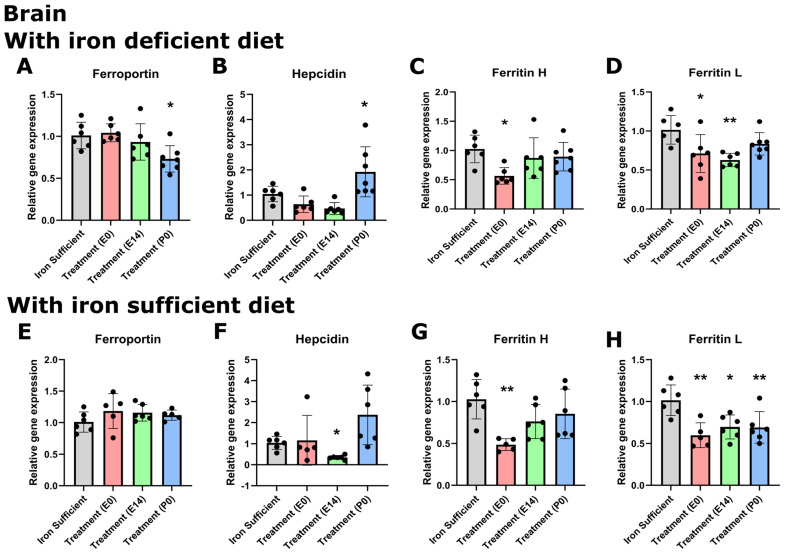
Expression of ferroportin, hepcidin, and ferritin H + L genes in the brain on P70 after maternal feeding an Fe-sufficient diet or an ID diet added with treatment with a single injection of FDI on E0, E14, or P0 (**A**,**E**). (**B**,**F**) Hepcidin expression is also only scarce in the CNS compared to that of the liver in normal-fed rats. A slightly higher expression is seen in rats of mothers fed with iron from P0. (**C**,**G**) Ferritin H is lower in the CNS in the E0 groups. (**D**,**H**) Ferritin L is slightly lower on E14 in the ID-fed groups and in the E0 group fed the iron-containing diet. Data are presented as mean ± SD with each sample marked as individual black dots (n = 5–8). * *p* < 0.05, ** *p* < 0.01.

**Figure 8:**
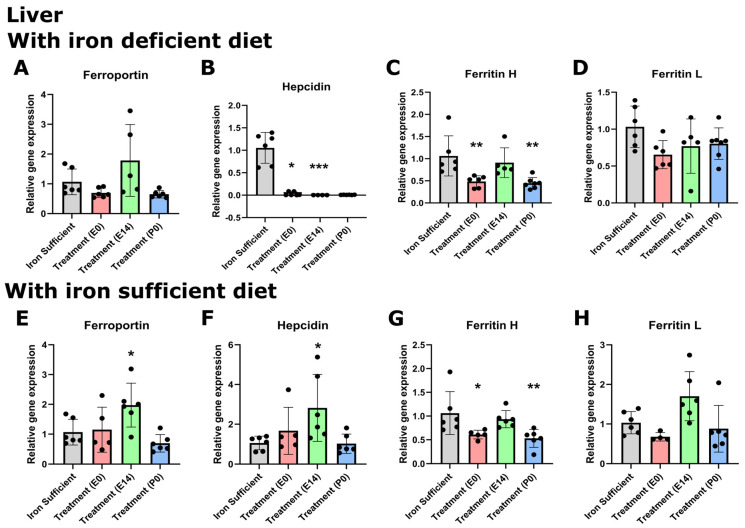
Expression of ferroportin, hepcidin, and ferritin H + L genes in the liver on P70 after maternal feeding a Fe-sufficient diet or an ID diet added with treatment with a single injection of FDI on E0, E14, or P0. (**A**,**E**) In the liver, ferroportin expression increases at E14. (**B**,**F**) Concerning hepcidin, expression is lower in treatment groups E0, E14, and P0 but normal (E0, P0) and higher (E14) when fed the iron-sufficient diet. (**C**,**G**) Ferritin H is lower in E0 and P0 groups fed either diet, whereas ferritin L (**D**,**H**) is unchanged in the liver. Data are presented as mean ± SD with each sample marked as individual black dots (n = 5–8). * *p* < 0.05, ** *p* < 0.01, *** *p* < 0.001.

While the changes in tissue metal concentration largely correlated with the type of postnatal diet at P70, we found that the pattern of gene expression did not change with the same diet dependency, with the important exception that the hepatic expression of hepcidin significantly lowered following ID (Figure 7 and Figure 8). Similar to that observed on P0, the gene expression of ferroportin in the brain was very low compared to that of the liver in the P70 rats. The cerebral expression among the various groups was insignificant except for a slightly lower expression in the P0 group. In the liver, ferroportin gene expression was higher in the E group (Figure 8A). The expression of hepcidin in the brain was very low in rats of mothers injected with FDI on E0 and E14, and higher in rats of mothers injected with FDI on P0 (Figure 7B). However, this could be considered of less significance as the expressional level of hepcidin was extremely vague in all brain samples, especially compared with those of the liver (CT values available from the corresponding author on request). When rats were returned to the Fe-containing diet after weaning, the expression of hepcidin conversely tended to rise to levels higher than that of control animals consistently fed the Fe-sufficient diet, although still at very low levels. In rats of mothers kept on the ID diet added with DFI while continuing the ID dietary regimen after weaning, the hepatic expression of hepcidin fell to levels in the three injection groups to an extent that hardly was measurable (Figure 8B). Concerning the ferritin transcripts in the brain and liver on P70, an overall tendency was that ferritin H transcripts were around 50% lower in the brain compared to the liver, whereas ferritin L transcripts were fifteen times more abundant in the liver. The ferritin H subunit in the brain was lowest in the E0 group injected with FDI in the brain (Figure 7C), and also lower in this group when fed the iron-containing diet (Figure 7G). In the liver, the ferritin H subunit was lower in the E0 and P0 groups fed either diet (Figure 8C,G). The cerebral expression of the ferritin L subunit was lower in the E14 group when fed the ID diet (Figure 7D), and lower in the E0 group when fed the iron-containing diet (Figure 7H). In the liver, ferritin L was significant only when fed an iron-sufficient diet (Figure 8D,H). 

## 4. Discussion

The brain is vulnerable to embryonic and gestational ID with risks of causing persistent neurotransmitter deficiencies, volume loss, and behavioral abnormalities [29,30,31,32,33,34]. Many of these adverse effects are preventable by iron supplementation, although abnormalities may persist [31]. In the present study, we aimed to evaluate if parenteral Fe supplementation with ferric derisomaltoside (FDI; formerly Fe isomaltoside 1000) [21,22] given to ID, pregnant female rats would be able to secure normal levels of Fe in the CNS in the offspring examined immediately after birth and in adulthood. For our study, we introduced a new model of gestational ID [35] where IIM was administered at different time points throughout the gestational period to pregnant rats with ID. IIM was effective in maintaining brain Fe concentration during gestation despite maternal ID, although the effect could not be sustained if the animals were kept on an ID diet after birth. Similarly, liver Fe was restored at P70 in animals receiving Fe-sufficient diet after birth, whereas they were depleted when ID was sustained.

Since hepcidin expression increases in the liver as part of the pro-inflammatory response and may impede erythropoiesis [36,37,38], we also examined its expression to exclude the possibility that ID was a result of inflammatory activity. Hepcidin levels decreased after ID in the liver, and the blood samples of the P70 rats revealed the expected anemia in ID groups. The leukocytes occurred in normal concentration showing that the rats were healthy and most likely without affections from infections. Interestingly, the ID rats had significantly higher concentrations of thrombocytes. This observation was also reported elsewhere and could rely on the fact that ID increases the differentiation of megakaryocytes and alters the phenotype of platelets [39].

The subcutaneously injected Fe bound to carbohydrate moieties in IIM is likely metabolized by the liver followed by insertion of Fe in transferrin and secretion of holo-transferrin into the blood plasma. While the ID mothers were healthy before, during, and after their pregnancy, pups that did not receive Fe treatment were hardly viable and died within three weeks after birth. This observation supports the theory of prioritized Fe acquisition during pregnancy, i.e., the first priority is to ensure fetal Fe levels, the second priority is the maternal hematocrit, and the third is the maternal Fe stores [40]. ID during pregnancy led to significantly lower levels of Fe in the brain and liver of newborn rats compared to levels in control pups but supplementing with IIM to the pregnant mother on E0 and E14 restored the cerebral Fe concentration. The expression of transferrin receptors on brain capillary endothelial cells in the developing brain is substantial, which suggests that the brain is capable of obtaining a higher uptake of Fe compared to other organs [41,42]. The capillaries of the brain are the only capillaries in the body that contain endothelial cells expressing transferrin receptors [9]. Genetic ablation of transferrin receptors leads to severe malformations in the developing neural tube and non-viable offspring [8], which supports the notion that cerebral Fe during development is of high priority and that the body attempts to secure Fe in the brain before many other organs [43,44]. 

Fe supplementation is in some countries recommended to all pregnant women from the beginning of gestation to reduce the risk of neurodevelopmental defects, but in general, recommendations differ across the world [45]. In animal models, reversion of ID needs intervention before the period of growth spurt and high myelination activity in the brain, since insufficient Fe supply in this stage of brain development could lead to irreversible defects [24,46,47]. Thus, both the Fe requirement and the time window in which it must be sufficient are strict, making efficient Fe supplementation via the diet difficult to control [48]. Adding to this difficulty is the poor intestinal absorption and side effects experienced by women taking oral Fe supplements [4]. A possible solution to this problem is to administer the Fe supplement parenterally at one single or multiple time points during gestation [35]. The theory behind this treatment regimen is that the large amounts of Fe contained in a bolus injection will be distributed in various tissues throughout the body while still being recruited (e.g., to the brain) should the need arise [22,23,24].

### 4.1. Fe and Fe-Related Genes in Offspring on P0

Cerebral ID on P0 was accompanied by a slight, yet significant, lowering of cerebral ferroportin gene expression, compared to control. This reduced expression was expected given the known downregulation of the ferroportin gene in ID [13]. The low expression of ferroportin could occur in the brain independently, at least in physiological conditions, without the post-translational regulation of hepcidin, which was suggested to occur in the heart with respect to the expression level of ferroportin [49]. 

The hepcidin gene expression level in the liver reflected well the expected response to sustained ID, i.e., manifest downregulation, whereas the Fe-sufficient diet was accompanied by an increase in animals treated at E0 and E14. Whether the brain expresses hepcidin is questionable [15,16], and the data of the present study indicate that the expression of hepcidin by the normal or ID brain is close to zero. Studies of the entire panel of non-neuronal cells in the mouse brain could not confirm any expression of the hepcidin gene, *hamp* [50]. The observations on ferroportin and hepcidin expression in the brain combined may illustrate an interesting dichotomy, where on the one hand IIM proved to be capable of restoring metal concentrations in the brain and liver, with the maintenance of these concentrations being dependent on the type of postnatal diet. On the other hand, the gestational ID and/or the different treatment regimens with IIM induced changes in gene expression that were less dependent on the postnatal diet, except for the gene expression of the master regulator of cellular Fe release, hepcidin, the levels of which were almost depleted in the liver upon chronic ID and despite IIM treatment. 

The two ferritin genes are translationally regulated [51], meaning that ferritin transcripts would not be expected to change following variations in the various dietary regimens. It was previously verified that dietary iron deficiency leads to the lowering of ferritin protein, whereas ferritin H and L mRNAs remain fairly constant [52,53]. Therefore, it was not surprising that the expression level of ferritin H and L mRNA subunits of the present study was generally unaltered in both brains and livers on P0. 

### 4.2. Fe and Fe-Related Genes in Offspring on P70

In the offspring examined on P70, cerebral Fe was significantly lower in dietary groups that were not supplied by the standard diet with normal Fe content. This observation was expected as the need for Fe in the postnatal period obviously was higher than could be covered by a single parenteral injection to the mother. Noteworthy, the Fe levels in these groups were around 50% lower than the normal fed rats in the brain, whereas this ratio was almost four-fold in the liver, which demonstrates the value of the preference for Fe-transferrin uptake by the brain due to the expression of transferrin receptors on brain capillary endothelial cells [9,12]. The capability of the offspring to achieve normal Fe levels in the brain and liver following substitution with a normal Fe content diet shows that the offspring were able to thrive despite the lower Fe content during gestation. The latter was also concluded when considering that the differences in the expression of ferroportin among the different groups in the brain and liver were non-significant. Like that of neonatal rats, the hepcidin expression of the P70 brain proved virtually undetectable and multifold lower than that of the liver. Despite the potential of rising expression in proinflammation, hepcidin mRNA did not increase in a mouse model with neurodegeneration and inflammation [54]. It cannot be excluded that hepcidin to some extent is being excreted from astrocytes in conditions with inflammation, but this statement awaits further evidence [55]. 

The liver exhibited the expected low expression of hepcidin following ID with values being almost zero both on P0 and P70. In contrast, the expression level in the liver was at least at the same level as the control group and sometimes even higher in rats that were reversed to the normal diet suggestive of a certain hyperplastic response to the iron-treatment [56].

As seen on P0, the brains and liver on P70 also were fairly constant in their expression levels of ferritin H and L mRNA. A prior study specifically addressed the relation between these isoforms in the adult rat brain, and it was also here concluded that the state of ID did not affect the amount of ferritin mRNA [53]. Ferritin L transcripts are more abundant than those of ferritin H [53], which was consistently also observed in the present study. 

### 4.3. The Effect of ID and Fe Therapy on Cu Levels 

The measured values of Cu and Zn are compatible with prior observations on the developing rat [54]. The significantly higher levels of Cu found in the liver of the Fe-sufficient group on P0 and also later in the liver on P70 are in good agreement with prior hypotheses and observations on the influence of iron status on the expression of Cu-transporters and Cu transport [57,58,59,60]. Like Fe, the abnormal regulation of Cu in the liver of the rats, when examined on P70, could not be rescued by a single parenteral injection of IIM, but rather relied on a combination of IIM and the Fe-containing diet. The gene expression of Cu transporting proteins in the placenta (CTR1, ATOX1, ATP7A) and liver (CTR1, ATOX1, ATP7B, ceruloplasmin) are unchanged in ID pregnant rats and also in the liver of their fetuses, which suggests that observed rise in hepatic Cu may not rely on increased expression among the Cu transporters [61,62]. 

On P70, the cerebral Cu level also appeared higher in groups of rats subjected to gestational ID when compared to controls. This is interesting, as Cu levels in the brain were normal at P0, and animals from E0 and E14 are fully Fe-complemented at P70 and also have normal Cu levels in the liver. Hence, our study indicates that ID in prenatal life—even though rescued before birth—triggers a delayed effect in brain-specific Cu metabolism that can be seen in adult life. The phenomenon of a change in gene expression to an environmental impact later, rather than early, in life has been described previously [63]. Possibly early ID mediates an altered expression in the brain of Cu transporters later in life.

### 4.4. The Effect of ID and Fe Therapy on Zn Levels 

On P0, the cerebral concentration of Zn was higher in the brains of the E0 group. Conversely, on P70 Zn was consistently lower in the groups with gestational ID no matter the current Fe status [60]. That the effect of ID on Zn is seen at P70, and not P0, may be due to the low level of peripheral Zn as indicated by the concentration of Zn in the liver at P0. This leads to reduced uptake of Zn everywhere in the body caused by the impact of Zn transporters following ID [60], which could impair neuronal formation, especially when combined with a lack of iron [63]. Significantly lower liver Zn levels were found in all groups at both P0 and P70 following ID, which is in accordance with prior observations [64,65] and may be explained by the fact that divalent metal ions compete for the same transporter (DMT1) in the gut. However, as we bypassed the intestinal route using parenteral Fe injection, the observed effects on hepatic Zn could be due to a possible impact on Zn transporters mediated by ID [66,67].

## 5. Conclusions

The main conclusions of this study are that ID during pregnancy in the rat is lethal for pups during weaning without supplemental Fe treatment immediately after birth. Secondly, the parenteral administration of IIM to pregnant females with ID secures normal levels of Fe in the brains of newborn pups, but when applied at later gestational ages, additional Fe to the offspring is needed to normalize cerebral and hepatic Fe. While the iron status in the rodent brain stem likely represents other brain regions that have similar developmental time frames, there could be regional differences, which should be pursued in future studies. Fe supplementation by IIM more readily restores cerebral Fe levels than liver Fe levels in pups. This observation likely relies on the selective expression of transferrin receptors of endothelial cells only in the brain. Lastly, ferroportin expression is slightly lower in ID. Ferroportin, and hepcidin in particular, are expressed only at low levels in the brain and are definitively much lower than seen in the liver. While the focus of this study was on measuring the mRNA expression of relevant iron-related genes, it should be noted that the concentration of ferroportin and ferritin proteins differ in iron deficiency and repletion due to their post-transcriptional regulation. Cu and Zn are changed even in adult rats, with cerebral and hepatic Cu being higher and the reverse with respect to Zn, hence clearly indicating the handling of these metals is affected by ID.

## Figures and Tables

**Table 1:** Representative data of female rats on P84 equal to their day of conception.

Pregnant Females	Weight	Hepatic Fe	Plasma Fe	HgB	Hepatic Cu	Hepatic Zn
	(g)	(µg/g)	(µg/g)	(mmol/l)	(µg/g)	(µg/g)
Iron-sufficient diet	221.3 ± 5.1	214.6 ± 22.5	460.3 ± 42.0	8.6 ± 0.4	2.7 ± 0.7	18.7 ± 5.5
Iron-deficient diet	237.4 ± 5.8	151.3 ± 24.2 *	461.5 ± 14.2	8.3 ± 0.5	2.9 ± 0.9	17.8 ± 5.2

The female rats of the iron-deficient group have iron deficiency without anemia. Cu, copper; Fe, iron; HGB, hemoglobin; Zn, zinc. Data are presented as mean ± SEM (n = 5–7). * *p* < 0.05.

**Table 2:** Growth and hematological parameters of offspring at P70.

Weight	HGB	MCV	MCHC	Reticulocytes	Leucocytes	Thrombocytes
(g)	(mmol/L)	(fL)	(mmol/L)	(×10^9^/L)	(×10^9^/L)	(×10^9^/L)
(1) Iron sufficient:						
275 ± 5.4	8.9 ± 0.2	59.1 ± 0.8	20 ± 0.1	227.7 ± 6.8	4.8 ± 0.9	615.5 ± 51.6
(II) Continuously iron-deficient diet with a single intramuscular injection of ferric derisomaltoside given on						
E0						
235.3 ± 4.7 **	3 ± 0.2 ***	39.7 ± 0.7 ***	11.3 ± 0.2 ***	1773 ± 121 ****	3.9 ± 0.8	891.4 ± 12.6 ***
E14						
258.1 ± 7.6	3 ± 0.2 ***	39.7 ± 0.3 ***	11.8 ± 0.1 ***	1380 ± 67.7 ***	3.4 ± 0.5	912.2 ± 50.9 ***
P0						
213.3 ± 9.7 ***	3.7 ± 0.1 ***	40.6 ± 0.4 ***	12 ± 0.2 ***	1591 ± 169.1 ****	5.7 ± 0.7	911.3 ± 43.9 ***
(III) Iron-deficient diet + Iron-containing diet after weaning. Single intramuscular injection of ferric derisomaltoside given on						
E0						
272 ± 3.5	8.8 ± 0.1	59.8 ± 0.3	19.6 ± 0.2	256.2 ± 12.1	2.8 ± 1.1	588.3 ± 56.1
E14						
264.6 ± 4.9	9.3 ± 0.3	59.8 ± 1.3	19.5 ± 0.3	275.8 ± 9.8	4 ± 0.4	586.7 ± 74.4
P0						
256 ± 8.1	9 ± 0.2	62 ± 1.0 *	19.3 ± 0.2	286.2 ± 19	4.8 ± 0.8	580 ± 35.6

MCV, Mean corpuscular volume; MCHC, Mean cell hemoglobin concentration; HGB, hemoglobin. Data are presented as mean ± SEM (n = 6–10). * *p* < 0.05, ** *p* < 0.01, *** *p* < 0.001., **** *p* < 0.001.

## Data Availability

The original contributions presented in the study are included in the article, further inquiries can be directed to the corresponding author.
